# Neuroprotective effect of novel cognitive enhancer noopept on AD-related cellular model involves the attenuation of apoptosis and tau hyperphosphorylation

**DOI:** 10.1186/s12929-014-0074-2

**Published:** 2014-08-06

**Authors:** Rita U Ostrovskaya, Yulia V Vakhitova, Uliyana Sh Kuzmina, Milyausha Kh Salimgareeva, Liana F Zainullina, Tatiana A Gudasheva, Vener A Vakhitov, Sergey B Seredenin

**Affiliations:** 1 Zakusov Institute of Pharmacology RAS, Baltiyskaya 8, Moscow, 125315, Russia; 2 Institute of Biochemistry and Genetics Ufa Scientific Centre RAS, Prospect Oktyabrya, 71, Ufa, 450054, Russia

**Keywords:** Alzheimer’s disease, Noopept, Beta-amyloid, Tau phosphorylation, Neurites outgrowth

## Abstract

**Background:**

Noopept (N-phenyl-acetyl-L-prolylglycine ethyl ester) was constructed as a dipeptide analog of the standard cognition enhancer, piracetam. Our previous experiments have demonstrated the cognition restoring effect of noopept in several animal models of Alzheimer disease (AD). Noopept was also shown to prevent ionic disbalance, excitotoxicity, free radicals and pro-inflammatory cytokines accumulation, and neurotrophine deficit typical for different kinds of brain damages, including AD. In this study, we investigated the neuroprotective action of noopept on cellular model of AD, Aβ_25–35_-induced toxicity in PC12 cells and revealed the underlying mechanisms.

**Results:**

The neuroprotective effect of noopept (added to the medium at 10 μM concentration, 72 hours before Аβ_25–35_) was studied on Аβ_25–35_-induced injury (5 μM for 24 h) in PC12 cells. The ability of drug to protect the impairments of cell viability, calcium homeostasis, ROS level, mitochondrial function, tau phosphorylation and neurite outgrowth caused by Аβ_25–35_ were evaluated.

Following the exposure of PC12 cells to Аβ_25–35_ an increase of the level of ROS, intracellular calcium, and tau phosphorylation at Ser396 were observed; these changes were accompanied by a decrease in cell viability and an increase of apoptosis. Noopept treatment before the amyloid-beta exposure improved PC12 cells viability, reduced the number of early and late apoptotic cells, the levels of intracellular reactive oxygen species and calcium and enhanced the mitochondrial membrane potential. In addition, pretreatment of PC12 cell with noopept significantly attenuated tau hyperphosphorylation at Ser396 and ameliorated the alterations of neurite outgrowth evoked by Аβ_25–35_.

**Conclusions:**

Taken together, these data provide evidence that novel cognitive enhancer noopept protects PC12 cell against deleterious actions of Aβ through inhibiting the oxidative damage and calcium overload as well as suppressing the mitochondrial apoptotic pathway. Moreover, neuroprotective properties of noopept likely include its ability to decrease tau phosphorylation and to restore the altered morphology of PC12 cells. Therefore, this nootropic dipeptide is able to positively affect not only common pathogenic pathways but also disease-specific mechanisms underlying Aβ-related pathology.

## Background

Alzheimer’s disease (AD) is the most common form of neurodegenerative disease, accompanied by age-related dementia, affecting 27 million individuals worldwide [1]. Mechanisms underlying the progression of late-onset AD consist of a number of interacting events including excessive accumulation of amyloid, aberrant tau-protein phosphorylation, oxidative stress, chronic inflammatory conditions, excitotoxicity, disruption of neurotrophine signaling, impairments in cytoskeleton stability and axonal transport, synaptic and neuronal loss [2]. Pharmacological treatment of AD currently involves cholinesterase inhibitors and NMDA receptor antagonists. Unfortunately, according to most investigators therapeutics of both these groups provide mainly symptomatic benefits without counteracting the progression of the disease [3].

Drug research in the last decade has attempted to develop disease-modifying drugs hopefully able to delay the onset or counteract the progression of AD. Strategies targeting at Aβ pathology include decreasing of Aβ production, preventing aggregation of Aβ into amyloid plaques, stimulating clearance of Aβ. Neither inhibitors of β-secretase or γ-secretase, nor stimulators of α-secretase have demonstrated satisfactory potency combined with low toxicity. Drugs targeting tau-protein are known to be divided into several groups: modulators of tau phosphorylation, inhibitors of tau-phosphorylating kinases (e.g. glycogen-synthase-kinase-3β, cyclin-dependent kinase-5, p70-S6-kinase) and compounds that prevent tau aggregation and misfolding [4].

AD is a complex multifactorial pathology, including multiple cycles and subcycles of self-amplifying neurodegenerative process [5],[6]. Monotherapy targeting single steps in this complicated cascade may explain disappointments in trials with agents affecting only one chain of this “circulus vituosus“. So it would be advantageous to explore the possibilities of novel multi-target therapy, aimed to affect different disease-related mechanisms, resulting in additive or synergic therapeutic responses [7].

Neuropeptides have drawn special attention as potential multitarget drugs because of their high biological activity (several orders higher than that of nonpeptide ones), availability of several recognising sites supposed to be complimentary to various targets, the ability to interact with different signal molecules, and minimal side effects. However, their usage as drugs is hindered by the poor blood–brain barrier penetration and low biological stability [8].

Design of dipeptides is one of the promising approaches taking into account high biological stability of these short molecules and presence of specific ATP-dependent transport systems for di/tripeptides in the intestine (PEPT1) and in the blood–brain barrier (PEPT2) [9]. This provides a basis for brain availability of dipeptides in case of systemic route of administration, including peroral one.

**Figure 1:**
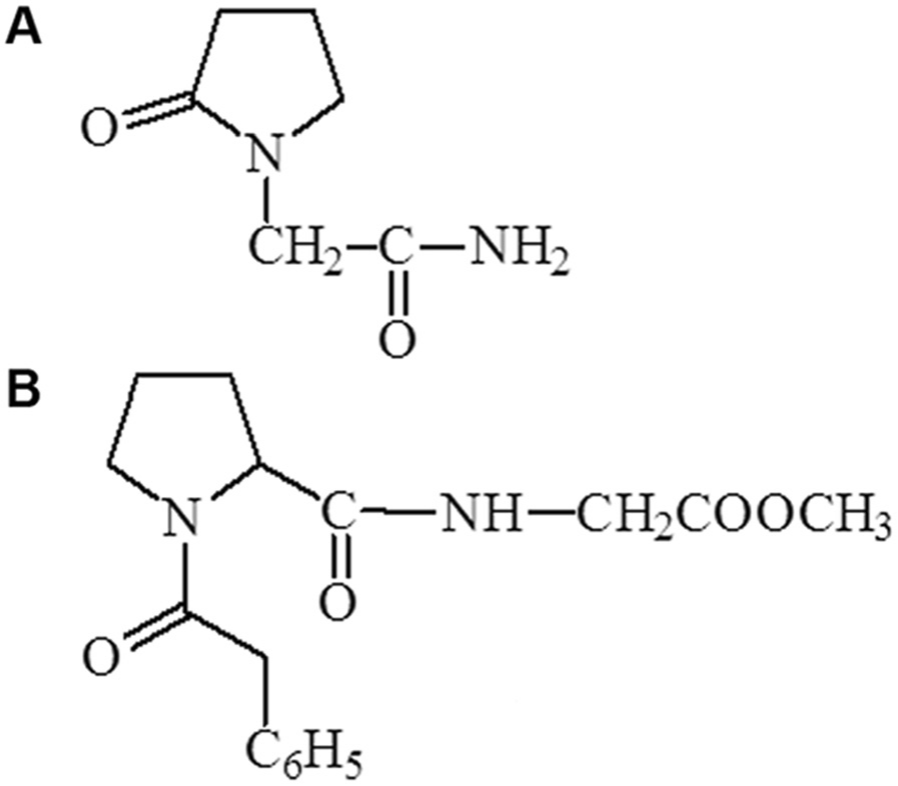
**Chemical structures of piracetam and noopept.** The structural similarity of piracetam **(A)** to noopept **(B)**. Both molecules contain pyrrolidine ring, acylated nitrogen in this ring, amide moiety and the fragment of glycine.

Original approach to the design of active dipeptides is being developed for many years at V.V. Zakusov Institute of Pharmacology. Searching for dipeptides with cognitive enhancing activity Gudasheva et al. based on the idea to get the structures conformationally close to piracetam as a standard cognition enhancer [10]. This drug-based peptide design led us to the series of acyl-prolyl-containing dipeptides possessing pronounced cognitive enhancing and neuroprotective activities [11]. Noopept (N-phenyl-acetyl-L-prolylglycine ethyl ester, GVS-111, Noopept®) (Figure 1) was chosen from this series because of its pronounced nootropic activity [12], high bioavailability for brain tissues in case of peroral administration [13] and specificity of its mechanism of action [14]. Noopept demonstrated wide spectrum of cognition improving effects [15] as well as pronounced neuroprotective activities both in vivo [15] and in vitro conditions [16]. Compared to piracetam noopept produces a cognition enhancing effect at much lower concentrations and demonstrates activity over a wider range of cognition disturbances and neuronal damages [17]. Noopept showed effectiveness in several animal models of AD: olfactory bulbectomy [18], administration of amyloid into Meinert nucleus [19] and intracerebroventricul administration of diabetogenic toxin streptozotocine [20]. Moreover, the experimental data on cognitive improving effect of noopept have been confirmed in clinic (Phase III and postregistration trials) demonstrating beneficial effect on cognitive functions in patients with MCI of cerebro-vascular or posttraumatic origin [21], and in particular in patients with amnestic form of MCI carrying *APOE ε*4^+^ allele [22]. Taken together these findings prompted us to continue the investigation of noopept on the cellular AD-related model. In the present study we investigated the protective effect of noopept against Аβ_25–35_-mediated damage of PC12 cells, measuring the cellular viability, apoptosis, intracellular Ca^2+^, ROS, mitochondrial membrane potential, tau protein phosphorylation level and neurite outgrowth. Aβ_25–35_ fragment was used as a peptide mimicking several of the toxic effects of the full-length amyloid-β peptide and therefore widely exploiting in both in vitro and in vivo Alzheimer’s disease models [23].

## Methods

### Cell cultures and treatments

PC12 cells were cultured routinely at 37°C in DMEM medium, supplemented with 10% fetal bovine serum (FBS), 5% horse serum, 2 mM L-glutamine, 50 μg/ml gentamicin. To induce PC12 differentiation, NGF (50 ng/ml; Sigma-Aldrich Inc., USA) was added to the DMEM containing 1% FBS, followed by a 5-day incubation. Differentiated PC12 (dPC12) cells were pretreated with noopept at concentration of 10 μM for 72 h, then cells were rinsed with the medium and exposed to amyloid-β-peptide (Аβ_25–35_, 5 μM; Tocris Bioscience, UK) for 24 h. Untreated cells were used as control.

### Cell viability and apoptosis measurements

Cell viability was determined by conventional MTT assay. dPC12 cells were plated in 24-well plates with 500 μl DMEM medium at the density of 1 × 10^4^ cells/well. After treatment with noopept (10 μM) for 72 h followed by Аβ_25–35_ (5 μM) for 24 h, cells were incubated with 200 μl MTT solution (0.5 mg/ml) at 37°C for additional 4 h. Thereafter the cells were solubilized with 200 μl dimethylsulfoxide. After mixing for 10 min absorbance was measured at 540 nm using the microplate spectrophotometer (EnSpire® Multimode Plate Reader; Perkin Elmer, USA). Cell viability was expressed as the percentage to cell viability in control. Flow cytometry analysis was used to identify the apoptotic cells. dPC12 cells (5 × 10^4^) in 6-well plates were treated as described above. Cells were harvested, washed out with cold phosphate-buffered saline (PBS) and stained with the Annexin V/PI (Annexin V-FITC Kit, Beckman Coulter Inc., USA) according to the manufacturer’s instructions. The data were processed using the FCS Express 4 software (*De novo* Software, USA) and the Cytomics FC 500 flow cytometer (Beckman Coulter, USA).

### Measurement of intracellular Ca^2+^

After incubation with noopept and Аβ_25–35_ dPC12 cells (1 × 10^4^ cells/well) were washed in Ca^2+^-free HBSS, containing 2.5 mM probenecid (Tocris Bioscience, UK). Then cells were loaded with 4 μM of Ca^2+^ indicator Fluo-4 AM and 0.02% pluronic acid (Invitrogen, USA) and incubated for 20 min at 30°C. Cells were washed out twice in buffer without dye, and incubated for further 15 min. The fluorescence of samples in 0.1 ml of buffer in new 96-well plates was monitored by the microplate spectrophotometer, using 485 nm excitation filter and 520 nm emission filter.

### Measurement of intracellular reactive oxygen species (ROS)

The generation of ROS was measured by the oxidative conversion of cell permeable 2,7-dichlorofluorescein diacetate (H_2_DCFDA; Invitrogen, USA) to fluorescent dichlorofluorescein. dPC12 cells (5 × 10^3^ cells/well) in 96-well plates were cultured for 72 h in 10% DMEM medium with noopept at concentrations of 10 μM. H_2_DCFDA was then added directly to the growth medium at a final concentration of 5 μM; cells were incubated for 1 h at 37°C. Cells were rinsed twice with PBS, placed in a fresh medium and treated with Аβ_25–35_ (5 μM) for 24 h. After this treatment cells were washed out with PBS. The plates were then read on the microplate spectrophotometer with 485 nm excitation and 535 nm emission wavelengths.

### Assessment of mitochondrial function

dPC12 cells were plated at a density of 5 × 10^3^ cells/well in 96-well plates. After treatment with noopept (10 μM) for 72 h and Аβ_25–35_ (5 μM) for 24 h changes in the mitochondrial membrane potential (MMP) were determined by incubating with 10 μM of JC-1 reagent (Invitrogen, USA) for 20 min at 37°C in the darkness. Then the cells were washed with PBS three times, and the fluorescent intensity was determined by microplate reader.

### Western blotting

dPC12 cells (5 × 10^4^ cells/per well) were treated as described above and after incubation the cells were harvested and suspended in lysis buffer (10 mM Tris, 1 mM EDTA, 1% SDS, pH 7.5). Protein concentrations were determined by the Bradford assay and equivalent amounts (10–15 μg) of total cellular proteins were separated by electrophoresis on a 12% SDS - polyacrylamide gel. Proteins were transferred to PVDV membrane and probed with anti-p-tau (Ser396; 1:800 v/v; Abcam, England) antibodies. After incubation with horseradish peroxidase–conjugated secondary antibody (1:10000; BioRad, Hercules, USA), immunoblots were developed using “Pierce ECL Western Blotting Substrate” (Thermo Scientific, USA). Membranes were stripped off and reprobed with anti-β-tubulin antibody (1:2000 v/v; Cell Signaling, USA) for loading control. Immunoblots were quantified by densitometry (ImageJ, http://rsbweb.nih.gov/ij/). Data were normalized to β-tubulin and the corresponding control was taken as 100%.

### Immunocytochemistry and morphometry

dPC12 cells (1 × 10^4^ cells/well) were plated onto poly-L-lysine coated coverslips in 24-well plates. After the treatment, cells were fixed with 4% paraformaldehyde, permeabilized with 0.2% Triton X-100 for 10 min and stained with mouse monoclonal antibody to neuron specific beta III tubulin (1:100; Abcam, England), followed by AlexaFluor 488-conjugated secondary antibody (1:1000; Invitrogen, USA). Coverslips were then incubated with Hoechst 33258 (1 μg/ml) for 5 min at room temperature. After washing out with PBS, samples were mounted in Mowiol 4–88 based media (Sigma, USA). Fluorescent images were obtained with Axio Imager (Carl Zeiss, Germany) microscope with acquisition settings at the maximal resolution (1024 × 1024 pixels) with 20x objective. Morphological analysis of cells (the number of neurites per cell and average neurite length) was carried out with Sholl analysis (Sholl analysis plug-in for ImageJ, http://rsbweb.nih.gov/ij/). Cells with at least one visible process equal to or greater than one cell body were considered as positive for neurite formation. All neurites of individual PC12 cell were traced, and the number of pixels was automatically converted to micrometers. Comparison of the number of processes between the experimental groups was carried out at a distance of 55 μm from the body of the cell. 50 randomly chosen cells were photographed and examined in each of three coverslips for each experimental condition. Results were obtained from three independent experiments.

### Statistical analysis

Each of the above listed parameters was measured in 3 to 5 independent experiments with 3–5 technical replicates per separate experiments. Statistical analysis was performed by one-way analysis of variance (ANOVA) followed by Turkey’s post-hoc test (Statistica v.6.0., StatSoft Inc., OK, USA). Data represent the mean ± SEM. A difference was considered statistically significant if the *p* < 0.05.

## Results

### Effect of noopept on cell viability and apoptosis in Aβ_25–35_-treated PC12 cells

**Figure 2:**
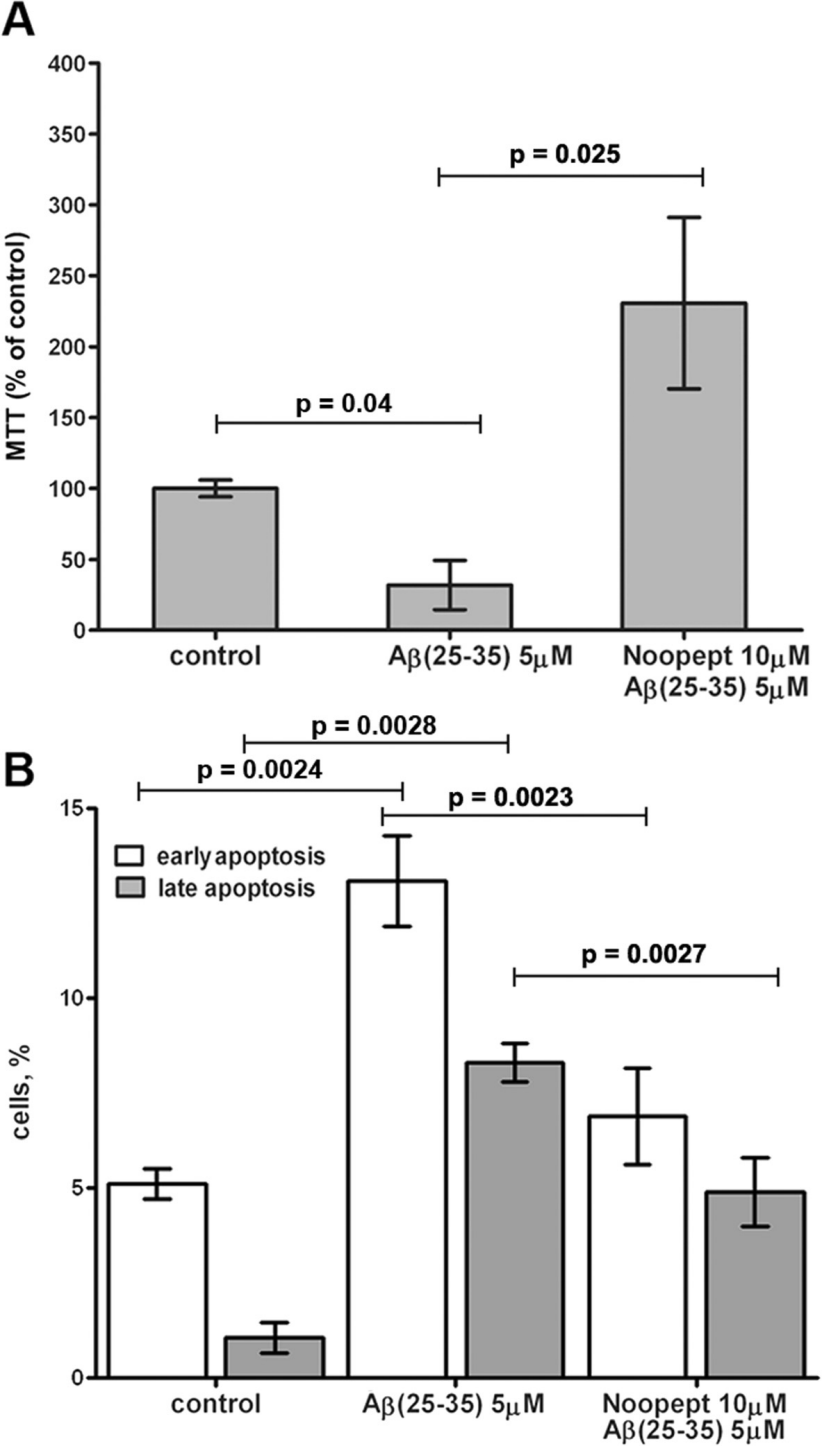
**Prevention of Aβ**_**25–35**_**- induced cytotoxicity by noopept. (A)** The cells were pre-treated with noopept (10 μM) for 72 h before exposure to 5 μM of Аβ_25–35_ for 24 h. Cell viability was determined by MTT assay. Data are expressed as means ± SEM. Five independent experiments were carried out in triplicate. **(B)** Apoptosis was assessed by double staining of cells with Annexin-V-FITC and propidium iodide. The bar chart represents the percentage distribution of apoptotic cells. Data are expressed as means ± SEM. Three independent experiments were carried out in triplicate.

A 24-h incubation of PC12 cells with Aβ_25–35_ (5 μM) decreased cell viability measured by MTT-test up to 32 ± 17.35%. Exposure of PC12 cells to noopept (10 μM, 72 h) significantly (p = 0.025) reduced cell death caused by Aβ_25–35_, increasing the cell viability to 230 ± 60.45% (Figure 2A). Therefore exposure of PC12 cells to noopept (10 μM, 72 h) not only attenuated the cytotoxic effect of Aβ_25–35_, but significantly (by about twofold comparing to intact control) increased the cell viability.

Apoptosis was quantified by double staining of cells with Annexin-V/PI (Figure 2B) to distinguish healthy PC12 cells (Annexin V-negative, PI-negative) from early apoptotic cells (Annexin V-positive, PI-negative) and late apoptotic cells (Annexin V-positive, PI-positive). Annexin V/PI staining revealed an increase in the percentage of early and late apoptotic cells from 5.1 ± 0.4 and 1.1 ± 0.4 in the control group to 13.1 ± 1.2 and 8.3 ± 0.5 respectively following incubation with Aβ_25–35_. Pretreatment of PC12 cells with noopept (10 μM for 72 h) prior to Aβ_25–35_ exposure, significantly decreased the percentage of Annexin V +/PI – (up to 6.9 ± 1.3; p = 0.0023) and Annexin V +/PI + cells (up to 4.9 ± 0.9; p = 0.0027), thus demonstrating the normalizing drug effect on early as well as on late apoptotic events.

### Effect of noopept on Ca^2+^ level, ROS production and mitochondrial membrane potential

**Figure 3:**
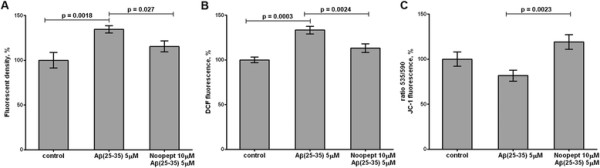
**Effect of noopept on Аβ**_**25–35**_**-evoked disturbances of intracellular calcium level, ROS accumulation and mitochondrial function. (A)** Pre-treatment with noopept reduces the rate of intracellular calcium in PC12 cells exposed to Aβ. **(B)** Noopept diminishes Аβ_25–35_ - induced enhancement of reactive oxygen species generation. **(C)** Noopept exposure ameliorates the mitochondrial membrane potential of PC12 cells after Аβ_25–35_-caused stress. Results represent means ± SEM. The values were obtained from three independent experiments with five technical replicates (A) and from five independent experiments with four technical replicates (B and C).

It is well known that Aβ_25–35_-caused cell death is accompanied by the rise of Ca^2+^, ROS accumulation and mitochondrial membrane potential disturbance in different neuronal and neuron-like cells. Exposure of differentiated PC12 cells to Aβ_25–35_ resulted in a 25% elevation of [Ca^2+^]_I_, while noopept statistically significantly (p = 0.027) inhibited calcium rise (Figure 3A). By using of the ROS fluorescent dye H_2_DCF-DA we were able to show that Aβ_25–35_ caused a moderate increase in ROS level, which was abolished by noopept (p = 0.0024) (Figure 3B). The noopept ability to counteract the Aβ_25–35_-induced cytotoxicity was also assessed by monitoring of the changes in the mitochondrial membrane potential using fluorescent dye JC-1. When PC12 cells were incubated with Aβ_25–35_ (5 μM for 24 h) a reduction of MMP was detected. Noopept was shown to protect the mitochondrial membrane potential against Aβ_25–35_ – induced mitochondrial disturbance (p = 0.0023) (Figure 3C). Taken together data obtained suggest that neuroprotective effect of noopept against beta amyloid neurotoxicity involves the limiting of oxidative stress, calcium disregulation and mitochondrial dysfunction.

### Noopept decreased tau phosphorylation induced by Aβ_25–35_

**Figure 4:**
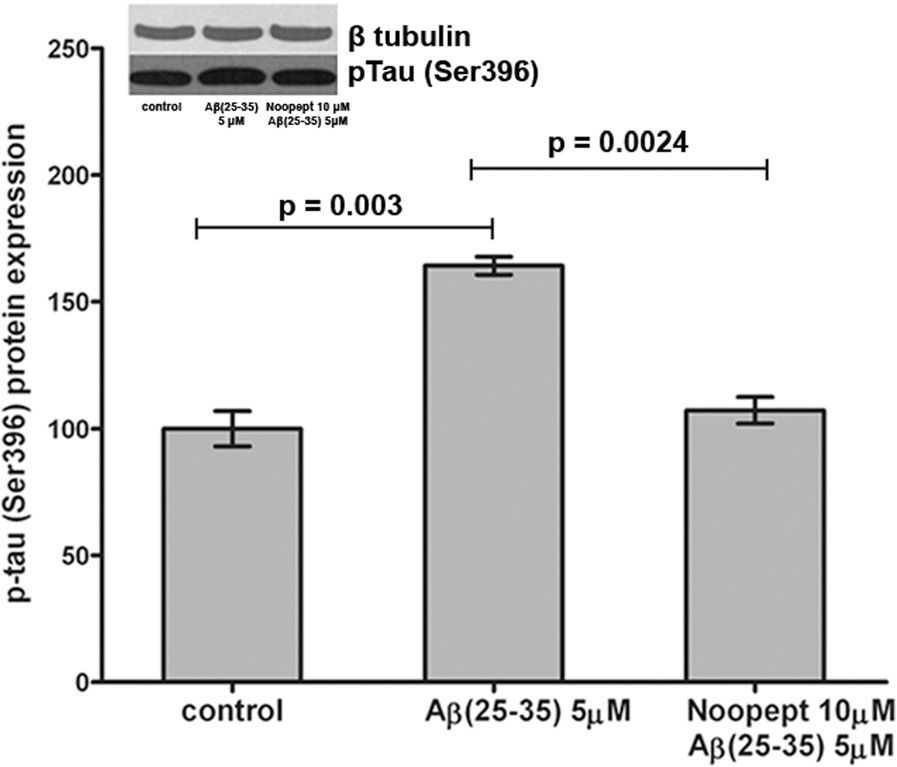
**Noopept decreases the tau phosphorylation induced by Аβ in PC12 cells.** Western blot analysis and graphs showed the changes in the content of the phosphorylated tau (Ser396) in PC12 cells pre-treated with noopept following by Аβ_25–35_ incubation. Densitometry values were normalized using the β-tubulin as internal control and expressed as means ± SEM. Four independent experiments were carried out using three replicate wells.

The effect of Aβ_25–35_ on tau protein phosphorylation level was measured by evaluating of the changes in immunoreactivity using anti-phospho-Ser396-tau antibodies. An increased level of tau phosphorylation at Ser396 was observed in the presence of 5 μM Aβ_25–35_, while the pretreatment with noopept caused the decline of p-tau Ser396 level (p = 0.0024) (Figure 4). Thus, the protective effect of noopept on Aβ_25–35_ toxicity apparently involves the attenuation of tau protein phosphorylation.

### Noopept ameliorates Aβ-induced impairment of PC12 cells morphology

**Figure 5:**
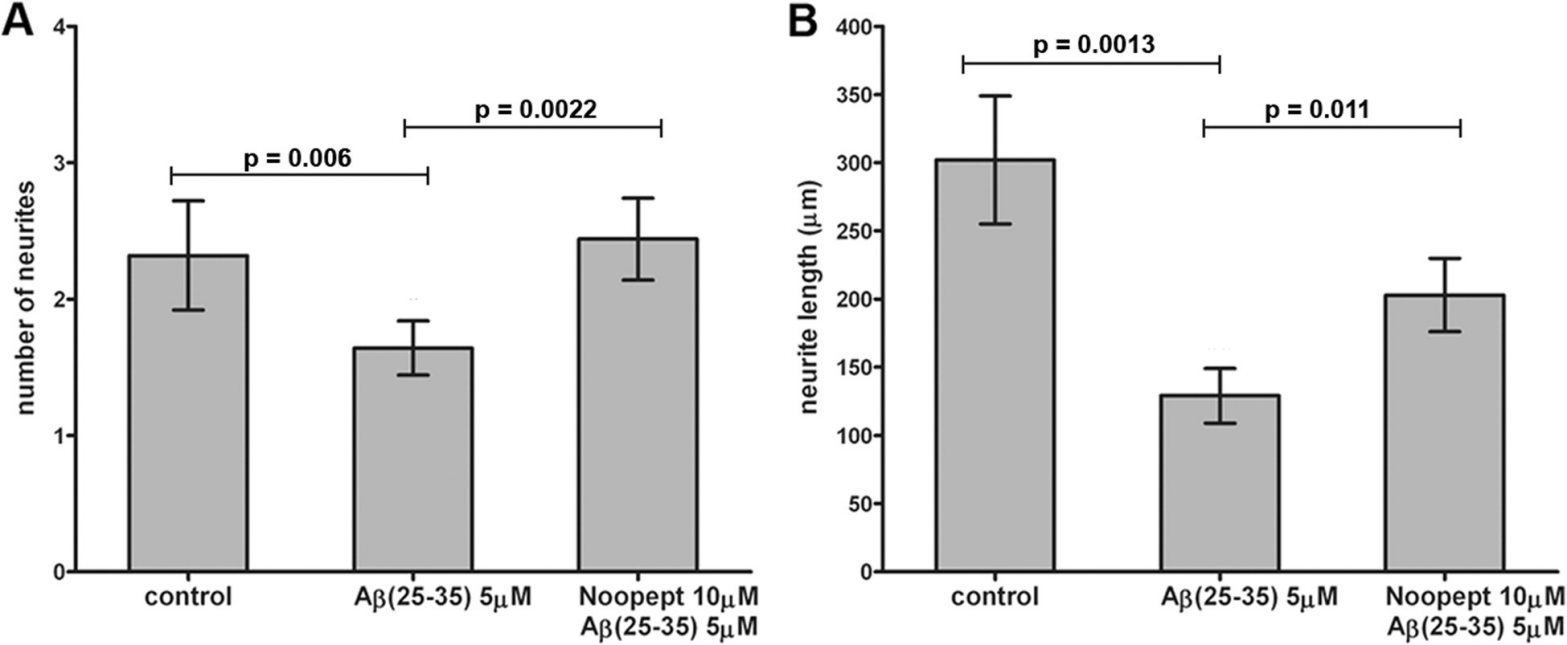
**Noopept protects the Аβ**_**25–35**_**- induced impairments of cells morphology. (A)** Quantification of number of IIIβ-tubulin - immunopositive neurites and **(B)** the average neurites length of PC12 cells after noopept pre-treatment following by Аβ_25–35_ addition. Data expressed as means ± SEM. Data from three coverslips (50 cells per coverslip) for each experimental group in three independent experiments were evaluated.

To further characterize the neuroprotective features of noopept we investigated the effect of the drug on morphology of differentiated PC12 cells. Quantification of neuritic complexity by determination of the average number and length of β-III-tubulin-immunopositive processes and neurites number at different distances from soma showed that PC12 cell treated with Aβ_25–35_ exhibited unfavorable changes in their cytoarchitecture. These changes were manifested in decreased number of neurites per cell (2.3 in control cultures versus 1.6 in Aβ-exposed cells), significantly reduced neurite length (from 302 μM up to 129 μM) (Figure 5A, B) and a decrease of neurites number with increasing distance from soma resulted in simplification of cells. The pretreatment of cells with noopept tended to overcome these detrimental effects of Aβ. In particular, the drug restored the number of neurites (2.44 versus 1.64; p = 0.0022) and increased their length compared to those in Aβ-treated group (from 129 μM up to 203 μM; p = 0.011) (Figure 5A, B). Overall the amount of longer neurites increased in noopept treated cells, compared to cells exposed to Aβ_25–35_ alone.

## Discussion

Present study revealed, for the first time, that the dipeptide cognition enhancing drug noopept protects differentiated PC12 cells against Aβ-mediated toxicity as evidenced by enhanced cell viability. While Aβ_25–35_ (5 μM) decreased cell viability, exposure of PC12 cells to noopept has not only overcome the depressing effect of amyloid on cells survival, but even increased it by about twofold compared to intact control. Our results further indicate that pre-treatment of the cells with noopept reduced the percentage of apoptotic cells observed following incubation with the Aβ_25–35_ peptide. Using Annexin V-FITC/PI double staining for the distinction of early- and late-apoptotic cells, we demonstrated that noopept attenuates both early and late apoptotic events induced by Aβ. Our findings of antiapoptotic effect of noopept against Aβ induced apoptosis are consistent with those obtained with this dipeptide in SH-SY5Y cells underwent to the toxic effect of another misfolded protein, α-synuclein amyloids [24].

Numerous in vivo and in vitro studies indicate that beta-amyloid triggers both common to different brain damages pathogenic pathways by inducing the rise of intracellular calcium level, reactive oxygen species production, alteration of mitochondrial function, and Aβ-specific signaling pathways resulted in increased tau phosphorylation [25]. Moreover, biochemical disturbances caused by Aβ are accompanied by substantial cytoskeleton abnormalities and consequently impaired axonal transport [26]. Particularly, prominent reductions of neurite outgrowth and neuritic elongation have been observed in different neuronal and neuron-like cells after oligomeric as well as fibrillar Aβ treatment [27],[28].

In this study, noopept was found to mitigate the intracellular calcium influx and excessive production of ROS, suggesting that the neuroprotective effects of the drug in this cellular model are probably associated with inhibition of Aβ-induced overload of calcium and antioxidant properties. Another mechanism involved in the neuroprotective action of noopept likely includes its ability to ameliorate mitochondrial dysfunction following Aβ_25–35_ exposure thereby interfering with mitochondrial apoptotic pathway.

These data are in accordance with our previous findings reporting neuroprotective action of noopept in various in vivo and in vitro studies. Noopept was shown to be able to normalize main secondary events by blocking the voltage dependent calcium channels [29], attenuating the neurotoxic effects of glutamate on granular cerebellar neurons [30], decreasing the glutamate release by cortical neurons [31]. Noopept significantly increased neuronal survival and prevented the accumulation of intracellular free radicals and apoptosis in experiments on cultured Down’s syndrome neurons [16]. The drug counteracted also the free radicals accumulation caused by α-synuclein on cultured neuroblastoma SH-SY5Y cells [24]. Interestingly, noopept was demonstrated to increase immunoreactivity to β-amyloid in mice with olfactory bulbectomy, considered as one of AD animal models [18].

Here we have shown for the first time that noopept can protect cells against Aβ-mediated toxicity by attenuating an increased tau phosphorylation at Ser396. Moreover, while Aβ-treated cells demonstrated decrease of neurites number and their length, noopept was shown to restore the number of neurites and significantly augment their processes length. It is known that extensively phosphorylated tau protein forms pathologic inclusions containing fibrillar aggregates were found in the brain of patients suffering from certain neurodegenerative disorders associated with dementias [32]. Tau protein is considered as one of the microtubules stabilizing proteins playing important role in facilitation of tubulin assembly into microtubules, thus contributing to the neurite outgrowth and maintenance of normal cellular morphology [33],[34]. Multiple studies provide evidence that the phosphorylation of tau at distinct serine/threonine residues by different protein kinases affects the ability of tau to promote microtubule polymerisation and stability [35]. Abnormally hyperphosphorylated tau possesses lower affinity for microtubules; it promotes the cytoskeleton rearrangements with consequent impairments of axonal transport and intracellular trafficking [36]. Neurite outgrowth of neuronal cells requires the assembly of tubulin into microtubules. The stability of microtubule network depends, at least in part, on the rate and extent of tau phosphorylation. Particularly, neurite outgrowth of neuronal and neuron-like cells was shown to correlate with the phosphorylation of tau at Ser262, Ser356, Ser396/404; these modifications reduce the ability of tau to bind to microtubules [37],[35]. A number of studies suggest that Aβ peptides under in vitro conditions can cause the increased phosphorylation of tau protein at different sites, thus provoking microtubules destabilization and cytoskeleton network degeneration [38],[26],[39]–[41]. Indeed, exposure of neuronal or neuron-like cells to the β-amyloid results in pronounced neurite retraction and reduced cell complexity [42]–[45] concomitant with a significant increase in tau phosphorylation at the Ser 396 whereas other serine/threonine sites – Ser199, Ser202, Thr205 and Ser404 show no significant alteration [46],[47]. Results from the present study suggest that abrogation of tau hyperphosphorylation at Ser396 by noopept eventually may play a role in restoration and even improvement of PC12 cell morphology. Neurite outgrowth promoting activity of noopept found in this cellular model, probably depends on drug’s ability to decrease the level of tau phosphorylation, thus affecting tau binding to microtubules. It should be mentioned that our previous experiments demonstrated noopept’ ability to increase the expression of NGF and BDNF in hippocampal and hypothalamic neurons in streptozotocin - intracerebroventricularly treated rats known to be an experimental model of sporadic AD [20]. PC12 cells express TrkA and respond to NGF by neurite outgrowth [48]. Findings of present study of noopept ability to exert antiapoptotic effect and to increase number and length of neuritis are in line with our supposition on the NGF involvement in above described effects of noopept on PC12 cells.

Recent studies provided evidence that both types of medicines currently used for AD treatment, NMDA receptor antagonists and AchE inhibitors, affect positively at least some of AD-related mechanisms. For example memantine was shown to inhibit the abnormal hyperphosphorylation of tau [49] and protected the neurons from Aβ-induced reduction of neurite outgrowth [50]. AchE inhibitor galantamine decreases the neuronal apoptosis induced by Aβ_25–35_, as well as membrane potential dissipation, suppressing the activity of caspase-9, caspase-12 and caspase-3 [51]. Results comparable to those obtained for noopept were observed for its conformationally related analog, piracetam. This cognitive enhancer attenuates the Aβ-caused alterations of mitochondrial membrane potential of PC12 cells and inhibited the negative effect of Aβ on neurite outgrowth [52].

Taken together findings obtained in this study suggest that noopept affects positively the core pathogenic mechanisms underlying the Aβ-mediated toxicity and provide new insights into the neuroprotective action of this drug and its possible beneficial effect in amyloid-related pathology. Further studies to confirm the neuroprotective effect of noopept against Aβ-induced neurotoxicity in AD animal model need to be conducted.

## Conclusions

Cognitive enhancer noopept exerts a protective effect against Aβ_25–35_-induced toxicity in PC12 cells. The protective ability of noopept most likely results from moderate suppression of oxidative stress and intracellular calcium influx, stabilization of mitochondrial function and reducing of apoptosis. Another possible mechanism by which this compound protects cells from amyloid toxicity may be related to the decrease of tau phosphorylation and, eventually, neurite stabilization and outgrowth.

## Abbreviations

Aβ: β-Amyloid

AchE: Acetylcholinesterase E

AD: Alzheimer disease

APOE: Apolipoprotein E

APP: Amyloid precursor protein

BDNF: Brain-derived neurotrophic factor

DMEM: Dulbecco’s modified Eagle’s medium

FBS: Fetal bovine serum

FITC: Fluorescein isothiocyanate

HBSS: Hank’s Balanced Salt Solution

H_2_DCFDA: 2’,7’-dichlorodihydrofluorescein diacetate

JC-1: 5,5’,6,6’-tetrachloro-1,1’,3,3’-tetraethylbenzimi- dazolylcarbocyanine iodide

MCI: Mild cognitive impairment

MMP: Mitochondrial membrane potential

MTT: 3-(4,5-dimethylthiazol-2-yl)-2,5-diphenyl-tetrazolium bromide

NGF: Nerve growth factor

NMDA: *N*-methyl-D-aspartate

PBS: Phosphate buffered saline

PEPT1: Peptide transporter 1

PEPT2: Peptide transporter 2

ROS: Reactive oxygen species

TrkA: Neurotrophic tyrosine kinase receptor type 1

## Competing interests

The authors declare that they have no competing interest.

## Authors’ contributions

SBS, RUO and TAG conceived the experiments. YVV and VAV designed the experiments. USK, MKS, LFZ performed the experiments and analyzed the data. RUO and YVV interpret the data and wrote the paper. All authors read and approved the final manuscript.
